# The factors affecting substance abuse relapse based on theory of planned behavior in male addicts covered by addiction treatment centers in Southern Iran

**DOI:** 10.1186/s12889-024-18733-1

**Published:** 2024-05-08

**Authors:** Mojtaba Sohrabpour, Amirhossein Kamyab, Asiyeh Yari, Pooyan Afzali Harsini, Ali Khani Jeihooni

**Affiliations:** 1 Noncommunicable Diseases Research Center, Fasa University of Medical Sciences, Fasa, Iran; 2 Faculty of Medicine, Fasa University of Medical Sciences, Fasa, Iran; 3 Department of Health Education and Health Promotion, School of Health, Hormozgan University of Medical Sciences, Bandar Abbas, Iran; 4 Department of Public Health, School of Health, Kermanshah University of Medical Sciences, Kermanshah, Iran; 5 Nutrition Research Center, Department of Public Health, School of Health, Shiraz University of Medical Sciences, Shiraz, Iran

**Keywords:** Relapse, Substance abuse, Theory of planned behavior, Addicts, Addiction treatment centers

## Abstract

**Background:**

Given the destructive nature of addiction and its relapse after quitting, the present study aimed to investigate the factors affecting substance abuse relapse based on the Theory of Planned Behavior (TPB) in male addicts covered by addiction treatment centers in Shiraz, Iran.

**Methods:**

This cross-sectional study was conducted on 400 male addicts covered by addiction treatment centers in Shiraz, Iran, in 2021–2022. The data collection tool was a researcher-made questionnaire. Data were analyzed using SPSS-22 software through descriptive statistical methods, linear regression, and binary logistic regression.

**Results:**

190 people (47.50%) were aged 31–40 years, 265 people (66.25%) were married, 224 people (56%) lived with their spouses, and 192 people (48 percent) had their first use at the age of 16–20. The substance respondents used were methamphetamine (59.5%), heroin (53%), opium (48%), and alcohol (40%). 138 people (34.5%) had their first place of consumption at friends’ houses (Tables 1 and 2).

342 people (85.5%) had a history of relapse, and 172 people (50.29%) had 1–5 relapses. Marital status, occupation, and income were among the demographic risk factors, and addicted friends and close relatives were among the behavioral risk factors for drug relapse among people with a history of relapse. Personal desire and the insistence of friends were also among the individual and interpersonal factors of drug use among participants. The regression results showed that the constructs of awareness, attitude, subjective norms, perceived behavioral control, and behavioral intention were predictors of drug relapse among addicts (*P* < 0.05).

**Conclusion:**

The current study’s findings indicate that among the behavioral risk factors for drug relapse in individuals with a history of relapse are addicted friends and close relatives, while marital status, occupation, and income are among the demographic risk variables. Among the individual and interpersonal factors influencing drug usage among participants were personal desire and friends’ insistence. Furthermore, the findings indicated that the TPB’s structures might be used to predict drug relapse in addicts.

## Background

Substance dependence is known as a chronic and recurrent brain disorder that is followed by compulsive seeking and consumption despite harmful consequences [1]. These harmful consequences, with physical, psychological, social, acute, and chronic aspects, lead to serious social problems such as crime, unemployment, family destruction, and inappropriate use of medical care [2]. The United Nations Office on Drugs and Crime (UNODC) in 2020 has raised new and serious warnings about the increased number of drug users. The 2020 UNODC report is far more alarming than the 2019 report, which compiled global data for 2017. According to the latest information from UNODC and based on regional reports, the total number of people aged 15–64 who have used drugs or stimulants at least once by the end of 2018 has increased by 30% compared to 9 years ago, reaching 269 million [3].

Addiction in Iran has also been growing in recent years. Due to its proximity to Afghanistan, as the world’s largest poppy and opium producer, Iran is the largest consumer of opium and other opium derivatives in the world [4, 5]. The total number of drug addicts announced in Iran in the second half of 2018 and the beginning of 2019 was 2,800,000, and the mean age of users is 24 years, of which 94% are male and 6% are female users [6]. In Shiraz City, which is one of the southern cities of Iran, addiction to substance and alcohol has been recognized as a major problem [7, 8]. To combat the pervasive drug usage issue in the nation, Iran has launched a number of initiatives and programs aimed at helping people kick their addiction [9, 10]. These programs include of support groups for addicts, rehabilitation facilities, and education and awareness campaigns [11]. Still, there are a lot of obstacles in the way of properly combatting drug misuse in Iran [12].

Addiction relapse is the biggest problem for recovered addicts [13]. A relapse is a state when an individual resumes their prior levels of alcohol or drug usage after losing motivation to cut back or refrain from using these substances [14]. Relapse, contrary to slip, is a planned condition in which the person does not have any intention to continue the recovery plan [15]. There are many factors affecting the relapse, including education, social or peer pressures, work-related stress, interpersonal issues, family problems, negative or false emotions or beliefs, place of residence, socioeconomic status, and some personality characteristics such as self-control [16, 17]. Therefore, the transition from the drug-using world to the drug-free world requires more and may be a period of serious and challenging struggles [18].

Many studies show the high prevalence of addiction relapse. In a study by Witkowitz’s, more than 80% of addicts return to drug use in less than 6 months after quitting [19]. A prospective study by Xie et al., which lasted for ten years, showed that 25% of completely recovered subjects relapsed during the first year of the study and the remaining 75% during the follow-up period [20]. The high prevalence of relapse indicates the insufficient effect of current addiction treatment methods. Studies show that countless factors, including individual, interpersonal, social, and public policy factors, are effective in starting, continuing, and returning to addiction after quitting. In a study by Deepti et al., friends played the main role in the relapse [21]. Afkar et al. believed that individual, family, occupational, and economic factors were the most important predictors of the relapse [22].

Considering the high prevalence of addiction and the high rate of its relapse on the one hand and ineffective intervention programs on the other hand, the need to address affecting factors at the ecological level and to use a planning framework based on evidence and theories to conduct relapse prevention interventions is seriously felt. In this regard, various models and approaches for the development, implementation, and evaluation of health education and promotion programs have been prepared by the scientists, one of which is the Theory of Planned Behavior (TPB). TPB is a suitable educational design framework and model for identifying needs in health education and designing behavior-change interventions. This theory is a social-cognitive model of value expectation, stating that intention is the main determinant of behavior. In this model, the intention itself is under the influence of three independent constructs: attitude, subjective norms, and perceived behavioral control. Attitude reflects a person’s positive or negative evaluation of performing a behavior. Subjective norms refer to the fact that perceived social pressures may cause a person to perform a certain behavior or not. And finally, perceived behavioral control is the perceived difficulty or ease of performing a specific behavior, which directly or indirectly affects the behavior.

The TPB shows that when people positively evaluate performing a behavior, believe that important ones think that the person should perform that behavior, and imagine that performing the behavior is under their control, they intend to do the behavior. In addition, in this theory, it is assumed that attitude, subjective norms, and perceived behavioral control are determined by their underlying beliefs [23]. Utilizing this theory, could effectively assess the factors affecting relapse. According to the nature of relapse and the factors affecting it. Given the importance of substance abuse relapse and its prevalence, factors influencing it, and the structures discussed in the TPB, the present study aimed to investigate the factors affecting substance abuse relapse based using the TPB in a group of male addicts covered by addiction treatment centers in Shiraz, Iran.

## Methods

This cross-sectional study was conducted on 400 male addicts covered by addiction treatment centers, which are designed to quit different kinds of substance addictions, in Shiraz, Iran, in 2021–2022. To collect data, after obtaining the necessary permits, the researcher referred to the addiction treatment centers, coordinated with the officials, and obtained informed written consent from the participants. The questionnaires were then provided to the participants.

### Data collection tool

The data collection tool was a researcher-made questionnaire designed by reviewing different studies [24–29], including three parts of demographic characteristics (age, education, marital status, occupation, income, place of residence, residence status, and monthly income), behavioral risk factors (questions like which drug did you use?; at what age did you use drugs for the first time?; have you ever slipped?; how many relapses did you do?; which of the following are the main reasons for relapse?), and TPB constructs (awareness, attitude, subjective norms, perceived behavioral control, and intention).

### TPB questionnaire

In this questionnaire, awareness was assessed by 15 questions on a 5-point Likert scale, ranging from 15 to 75. Attitude structure was measured by 10 questions on a 5-point Likert scale, ranging from 10 to 50. Each of the subjective norms and perceived behavioral variables were quantified using eight questions on a 5-point Likert scale, ranging from 8 to 40.

### Validity of the questionnaire

An item effect size more than 0.15 and a content validity ratio greater than 0.79 were used to evaluate the validity of the questionnaire. To determine the face validity of the tool, a list of compiled items was targeted by 40 patients (who were under methadone treatment) with demographic, economic, and social characteristics similar to those of the population. The content validity was determined based on the opinions of 10 experts on health education and health promotion, 1 psychiatrist, and 1 psychologist.

Using the Lawshe index, items higher than 0.56 were considered essential and kept for further analysis. Most of the items were above 0.70. Based on Cronbach’s alpha, the overall reliability was calculated to be 0.89. Also, the reliability of awareness, attitude, subjective norms, perceived behavioral control, and behavioral intention was calculated to be 0.82, 0.89, 0.89, 0.88, and 0.87, respectively.

### Sampling method

The present study aimed to investigate the effect of educational intervention based on TPB on substance abuse relapse in male addicts covered by addiction treatment centers in Shiraz. The sampling method in the analytical-descriptive stage was cluster sampling. In this way, after obtaining the necessary permits from the ethics committee of the University of Medical Sciences, the list of active addiction treatment centers in Shiraz was first received. Then, 8 centers were randomly selected. It should be noted that if someone did not want to participate in the study, substitute subjects were included in the study. According to previous studies, the relapse rate was 80% [27], which required 264 subjects to conduct the study with 95% confidence and 5% accuracy. In addition, by applying the cluster factor of 1.5, the sample size increases to 400.

$$N=\frac{{Z}^{2}1-aP(1-P)}{{d}^{2}}$$

### Inclusion and exclusion criteria

Inclusion criteria were addicts covered by addiction treatment centers, having no chronic physical or mental diseases according to the medical records, and obtaining informed written consent to participate in the study. The exclusion criterion was the inability to answer the questions.

### Data analysis

Data were analyzed using SPSS 22 software through descriptive statistical methods, linear regression, and binary logistic regression.

## Results

In this research, 400 addicts were examined under the coverage of addiction treatment centers in Shiraz. 190 people (47.50%) were aged 31–40 years, and 142 people (35.50%) had a high school education. 265 people (66.25%) were married, and 224 people (56%) lived with their spouses. 272 people (68 percent) live in the city, 268 people (67 percent) have an income of less than 20 million riyals, and 192 people (48 percent) had their first use at the age of 16–20. methamphetamine (59.5%), heroin (53%), opium (48%), and alcohol (40%). 138 people (34.5%) had their first place of consumption at friends’ houses (Tables 1 and 2).

**Table 1:** Demographic characteristic of the participants and their association with relapse

Variables	No relapse	Relapsed	*P*-value
Age			
≤ 20	0 (0)	12 (3)	0.99
21–30	15 (3.75)	80 (20)	0.56
31–40	32 (8)	158 (39.50)	0.82
41–50	7 (1.75)	70 (17.50)	0.57
≥ 50	4 (1)	22 (5.50)	0.44
Education			
Illiterate	4 (1)	18 (4.5)	0.32
Primary school	10 (2.5)	70 (17.5)	0.35
Secondary school	12 (3)	106 (26.5)	0.28
High school	20 (5)	122 (30.5)	0.17
College	12 (3)	26 (6.5)	0.54
Marital status			
Married	50 (12.50)	215 (53.75)	0.14
Single	2 (0.50)	93 (23.25)	0.001
Widowed	1 (0.25)	1(0.25)	1
Divorced	5 (1.25)	33 (8.25)	0.32
Employment			
Worker	12 (3)	56 (14)	0.76
Employed	10 (2.5)	12 (3)	0.001
Self-employed	26 (6.5)	224 (56)	0.65
Unemployed	10(2.5)	50 (12.5)	0.72
Place of residence			
City	40 (10)	232 (58)	0.38
Village	18 (4.5)	110 (27.5)	0.25
Income			
≤ 20 million Rials	30 (7.5)	238 (59.5)	0.001
20–50 million Rials	18 (4.5)	70 (17.5)	0.27
≥ 50 million Rials	10 (2.5)	34 (8.5)	0.18

**Table 2:** Frequency distribution of individual and environmental risk factors for the relapse

Variables	Number	Percentage
The age of onset of use		
≤ 15	52	13
16–20	192	48
21–25	92	23
26–30	64	16
Type of substance used		
Cigarette	318	79.5
Hashish	124	31
Alcohol	160	40
Grass	82	20.5
Opium	192	48
EX	12	3
Norgesic	10	2.5
Cocaine	18	4.5
Heroine	212	53
Crack	84	21
Methamphetamine	238	59.5
Methadone	196	49
Tramadol	132	33
Other	22	5.5
Place of the first relapse		
One’s home	128	32
Friends’ home	138	34.5
Party	52	10.5
Street or park	42	10.5
Other	40	10

The findings of the study showed that 92 people (23 percent), their fathers, 82 people (20.5 percent), and 68 people (17 percent) best friends had the most history of drug use (Table 3). 342 people (85.5%) had a history of relapse, and 172 people (50.29%) had 1–5 relapses (Table 4). Marital status, occupation, and income were among the demographic risk factors, and addicted friends and close relatives were among the behavioral risk factors for drug relapse among people with a history of relapse. Personal desire and the insistence of friends were also among the individual and interpersonal factors of drug use among study participants (Table 5).

**Table 3:** Frequency distribution of history of drug use in family and friends

	Number	Percentage
Father	92	23
Mother	4	1
Brother	82	20.5
Sister	3	0.75
Close relatives	66	16.5
Other relatives	41	10.25
The best friend	68	17
Other friends	44	11
Total	400	100

**Table 4:** Frequency distribution of relapse status

History of relapse		Number	Percentage
No		58	14.5
Yes		342	85.5
History of relapse	Number of relapse	Number	Percentage
Yes	1–5	172	50.29
	6–10	96	28.07
	≥ 10	74	21.64

**Table 5:** Behavioral, personal, interpersonal, and environmental risk factors for the relapse

	No relapse	Relapsed	*P*-value
Behavioral risk factors			
Addicted father			
No	52 (13)	256 (64)	0.28
Yes	6 (1.5)	86 (21.50)	0.34
Addicted mother			
No	57 (14.25)	339 (84.75)	0.42
Yes	1 (0.25)	3 (0.75)	0.25
Addicted brother			
No	42 (10.50)	276 (69)	0.48
Yes	16 (4)	66 (16.50)	0.18
Addicted sister			
No	58 (14.50)	339 (92.25)	0.15
Yes	0 (0)	3 (0.75)	0.31
Addicted friends			
No	46 (11.50)	286 (71.50)	0.24
Yes	12 (3)	56 (14)	0.02
Other friends			
No	54 (13.50)	302 (75.50)	0.18
Yes	4 (1)	40 (10)	0.07
Close relatives			
No	47 (11.75)	287 (71.75)	0.12
Yes	11 (2.75)	55 (13.75)	0.02
Other relatives			
No	56 (14)	303 (75.75)	0.36
Yes	2 (0.50)	39 (9.75)	0.47
Personal, interpersonal and environmental risk factors			
Curiosity			
No	50 (12.5)	300 (75)	0.27
Yes	8 (2)	42 (10.50)	0.35
Inclination			
No	57 (14.25)	264 (66)	0.18
Yes	1 (0.25)	78 (19.50)	0.001
Friends’ pressure			
No	50 (12.50)	284 (71)	0.14
Yes	8 (2)	58 (14.50)	0.001
Pleasure			
No	53 (13.25)	244 (61)	0.16
Yes	5 (1.25)	98 (24.50)	0.02
Accessibility			
No	56 (14)	240 (60)	0.21
Yes	2 (0.5)	102 (25.50)	0.03
Place of residence			
No	56 (14)	310 (77.50)	0.12
Yes	2 (0.5)	32 (8)	0.22
Family issues			
No	56 (14)	254 (63.50)	0.16
Yes	2 (0.5)	88 (22)	0.02
Job failure			
No	52 (13)	302 (75.50)	0.24
Yes	6 (1.50)	40 (10)	0.82
Mental issues			
No	47 (11.75)	230 (57.50)	0.16
Yes	11 (2.75)	112 (28)	0.10
Escapism			
No	48 (12)	260 (65)	0.28
Yes	10 (2.50)	82 (20.50)	0.24

The results showed that among the constructs of the TPB, the constructs of attitude and behavioral intention had the highest mean (Table 6). The regression results showed that the constructs of awareness, attitude, subjective norms, perceived behavioral control, and behavioral intention were predictors of drug relapse among addicts (*P* < 0.05) (Table 7).

**Table 6:** Mean score of awareness, attitude, subjective norms, and perceived behavioral control of participants

Constructs	Mean	SD	Score range
Awareness	7.22	1.18	0–15
Attitude	20.77	3.09	10–50
Subjective norms	14.23	2.65	8–40
Behavioural control	13.14	2.94	8–40
Behavioural intention	14.40	2.55	8–40

**Table 7:** Analysis of linear regression in predicting relapse prevention

Constructs	B	SE	β	95% CI of B	*P*-value
Awareness	0.061	0.027	0.152	0–014.118	0.032
Attitude	0.052	0.038	0.096	0–024.122	0.041
Subjective norms	0.041	0.042	0.085	0–018.104	0.038
Behavioural control	0.064	0.025	0.228	0–012.112	0.024
Behavioural intention	0.085	0.034	0.234	0–021.230	0.020

## Discussion

The aim of this study was to examine the impact of educational interventions based on the TPB on substance abuse relapse among male addicts receiving treatment in Shiraz, Iran. Our findings shed light on several critical aspects related to substance abuse and relapse.

Firstly, the study highlighted the early onset of substance use, with nearly half of the participants initiating drug consumption before the age of 20. In a study by Mirzaei et al. [30], the age of onset of use for 63% of participants was under 20 years of age. This underscores the necessity of implementing effective addiction prevention programs targeting young individuals, as interventions during this critical period could potentially mitigate the risk of future substance abuse.

Regarding the types of substances consumed, our results indicated cigarettes, opium, heroin, and crack as the most addictive substances consumed. Notably, individuals who engage in polydrug use were found to be at a higher risk of developing substance abuse disorders. Consistently, local surveys have shown that opium was the most common type of substance consumed in Iran [31]. Our findings align with previous research by Guindalini et al. highlighting the correlation between concurrent use of multiple substances and increased susceptibility to addiction [32].

Furthermore, familial and social factors emerged as significant contributors to relapse. The influence of family dynamics and relationships on addiction propensity has been well-documented, with familial drug use history serving as a particularly influential factor [33]. Consistent with existing literature, our findings underscored the role of family support and the detrimental impact of familial drug use patterns on individuals’ susceptibility to relapse [34]. Our findings are consistent with those of Coviello et al. [35], Habibi et al. [36], and Habibi, Basharat, and Wreder-Ferrer [37].

In terms of relapse patterns, participants reported experiencing relapse multiple times, with various factors contributing to this recurrence. These factors included association with addicted peers, psychological stressors, environmental triggers, familial rejection, and exposure to drug-related stimuli. In line with our finding, Hoseyni and Falahzade [38] stated the factors influencing the relapse are drug addict friends, mental-psychological pressures, returning to former places, unfortunate situations, being rejected by family and society, and being in touch with objects of drug use. Moreover, previous studies showed that a majority of the surveyed addicts had a history of relapse in the first year of quitting [39–41]. Considering that background factors such as friends, family’s financial condition, personality, etc. play a decisive role in addiction relapse, and on the other hand, most quitted addicts do not correct their previous underlying factors, the possibility of relapse is highly expected.

Furthermore, socio-economic factors such as unemployment and low income emerged as significant risk factors for relapse. Economic instability and social marginalization can exacerbate vulnerabilities to substance abuse, emphasizing the importance of holistic support systems and socio-economic empowerment initiatives in addiction recovery efforts [42]. From the addict’s point of view, socializing with addicted and deviant friends has been described as the most important interpersonal factor in addiction relapse. However, unemployment, poverty, and inappropriate treatment by family members are among the other factors [43].

Individual-level factors, including personal pleasure and desire, were identified as influential determinants of relapse. This highlights the subjective nature of addiction and the pivotal role of individual motivations and cravings in driving substance use behaviors. Our results are consistent with the results of previous studies [44, 45]. Allahverdipour et al. [46] found the sense of curiosity and gaining pleasure as the most important causes of the tendency towards drugs. Conclusively, prevention programs must address these intrinsic factors by promoting healthier coping mechanisms and enhancing individuals’ resilience against cravings and temptations.

The findings also underscored the predictive power of TPB constructs in understanding and mitigating substance abuse behaviors. Constructs such as awareness, attitude, subjective norms, perceived behavioral control, and behavioral intention were identified as significant predictors of substance abuse and relapse. Additionally, educational interventions targeting these constructs have shown promise in promoting negative attitudes towards drug use and enhancing individuals’ ability to resist social influences and impulsive behaviors. Consistent with the present study, the results of studies by Moeini [47], Orbell [48], Olds [49], and Moan [50] have shown that models such as TPB are good predictors of unhealthy behaviors. Other studies have shown that training subjective norms could increase the subjects’ participation in changing their behavior [51].

In conclusion, our study contributes to the growing body of literature on substance abuse and relapse by elucidating the multifaceted determinants and predictive factors underlying addiction behaviors. By addressing socio-economic, familial, social, and individual-level factors, targeted interventions based on the TPB framework hold potential in mitigating substance abuse relapse and promoting long-term recovery among individuals battling addiction.

## Conclusion

The current study’s findings indicate that among the behavioral risk factors for drug relapse in individuals with a history of relapse are addicted friends and close relatives, while marital status, occupation, and income are among the demographic risk variables. Among the individual and interpersonal factors influencing drug usage among participants were personal desire and friends’ insistence. Furthermore, the findings indicated that the TPB’s structures can be used to predict drug relapse in addicts.

In other words, the disorders can be predicted based on the mentioned components. Therefore, conducting theory-based educational interventions in the future such as implementing the TPB in interventions conducted on addicts with a history of relapse could have a significant effect on reducing the intention of substance abuse in them.

## Data Availability

The datasets used and/or analyzed during the current study can be made available by the corresponding author on reasonable request.

## Abbreviations

TPB: Theory of Planned Behavior
BSSI: Beck Scale for Suicidal Ideation
